# Strict health-oriented eating patterns (orthorexic eating behaviours) and their connection with a vegetarian and vegan diet

**DOI:** 10.1007/s40519-018-0563-5

**Published:** 2018-08-29

**Authors:** Anna Brytek-Matera, Kamila Czepczor-Bernat, Helena Jurzak, Monika Kornacka, Natalia Kołodziejczyk

**Affiliations:** 1 Katowice Faculty of Psychology, SWPS University of Social Sciences and Humanities, Technikow 9, 40-326 Katowice, Poland; 2 Interdisciplinary Doctoral Studies, Wroclaw Faculty of Psychology, SWPS University of Social Sciences and Humanities, Wroclaw, Poland; 3 Faculty of Psychology, SWPS University of Social Sciences and Humanities, Warsaw, Poland

**Keywords:** Orthorexia nervosa, Vegetarian diet, Vegan diet, Eating behaviours

## Abstract

**Purpose:**

Although research on vegetarianism is becoming more prevalent, to date, only a few research has been conducted on relationship between vegetarian diet and orthorexia nervosa (ON). The objective of the present study was to examine the orthorexic dietary patterns and eating behaviours among individuals following a vegetarian, vegan, and meat diet. We examined the moderating role of ethical and health reasons for following a meat-free diet on the relation between vegan versus vegetarian diet and eating behaviours and ON. The study aimed to determine the predictors of ON in individuals with differential food preferences.

**Methods:**

Seventy-nine individuals following a meat-free diet and 41 individuals following an omnivore diet completed the EHQ and the TFEQ-R18.

**Results:**

Our findings indicated that individuals following a vegan diet showed a higher level of knowledge of healthy eating than those who followed a vegetarian diet and those who followed an omnivore diet. Participants maintaining a vegan diet for health reasons were more likely to have greater knowledge about healthy eating. Cognitive restraint was a predictor of ON among a sample following a meat-free diet.

**Conclusions:**

Our results could contribute to identify potential risk factors for strict health-oriented eating patterns and to gain a better insight into ON.

**Level of evidence:**

Level V, descriptive study.

## Introduction

Vegetarianism is defined as the practice of abstaining from eating meat [1] based mainly on ethical, but also health-related, aspects [2]. In the literature, one can find a hypothesis that orthorexic eating behaviour might appear more often among vegetarians than among people without specific dietary habits [3, 4]. However, only a few studies have explored this hypothesis [2, 5–8]. Moreover, the literature on the link between a vegetarian diet and orthorexia seems to lack consensual results, and research assessing what feature of orthorexic or maladaptive eating behaviour might be linked to specific vegetarian eating habits is still missing.

Orthorexia nervosa (ON) is defined as a fixation on health-conscious eating behaviour [9]. The first (formal) diagnostic criteria was developed by Moroze et al. [10]. Recently, Dunn and Bratman [11] proposed more detailed classification criteria (Table 1).

**Table 1:** Classification criteria for orthorexia nervosa by Dunn and Bratman [11]

Criterion A: Obsessive focus on “healthy” eating, as defined by a dietary theory or set of beliefs whose specific details may vary; marked by exaggerated emotional distress in relationship to food choices perceived as unhealthy; weight loss may ensue as a result of dietary choices, but this is not the primary goal. As evidenced by the following:
A1. Compulsive behaviour and/or mental preoccupation regarding affirmative and restrictive dietary practices believed by the individual to promote optimum health
A2. Violation of self-imposed dietary rules causes exaggerated fear of disease, sense of personal impurity and/or negative physical sensations, accompanied by anxiety and shame
A3. Dietary restrictions escalate over time, and may come to include elimination of entire food groups and involve progressively more frequent and/or severe “cleanses” (partial fasts) regarded as purifying or detoxifying. This escalation commonly leads to weight loss, but the desire to lose weight is absent, hidden or subordinated to ideation about healthy eating
Criterion B: The compulsive behaviour and mental preoccupation becomes clinically impairing by any of the following:
B1. Malnutrition, severe weight loss or other medical complications from restricted diet
B2. Intrapersonal distress or impairment of social, academic or vocational functioning secondary to beliefs or behaviours about healthy diet
B3. Positive body image, self-worth, identity and/or satisfaction excessively dependent on compliance with self-defined “healthy” eating behaviour

These reported criteria are new diagnostic criteria for ON, achieved after a critical review of published case histories, eating disorders professionals’ narrative descriptions, and numerous self-reports of orthorexia nervosa sending to Bratman’s website [11]. The previous criteria described by Bratman and Knight [12] have not been identified empirically, and it has not been empirically proven that they represent a co-occurring pattern of behaviours [13].

Although ON cannot be considered as a diagnostic category and still needs to be recognized as neither DSM-5 nor ICD-10 do not consider it as a syndrome, orthorexic behaviour can represent an important limitation in everyday life deeply affecting the quality of life. ON starts out as an innocent attempt to obtain optimum health through diet, but it finally leads to unintended negative consequences such as malnutrition, impaired social life, deterioration of the quality of life, and well-being [14, 15]. Diet becomes essential part of people’s thoughts and concerns and leads to dietary restrictions, excessive focus on food-related topics, lack of enjoyment of food, gaining control over food intake, rigid eating behaviours, and ritual actions involving food preparation [16–18]. Individuals with ON desire to improve self-esteem and self-realization through controlling food intake [19]. Sometimes, all behaviours may be associated with unintentional weight loss, with no desire to lose weight (losing weight is subordinated to ideation about healthy food). To sum up, ON include abnormal (compulsive) behaviours or mental preoccupations with dietary choices believed to promote optimal health, self-imposed anxiety, self-punishment, and escalating severe restrictions [20].

**Fig. 1:**
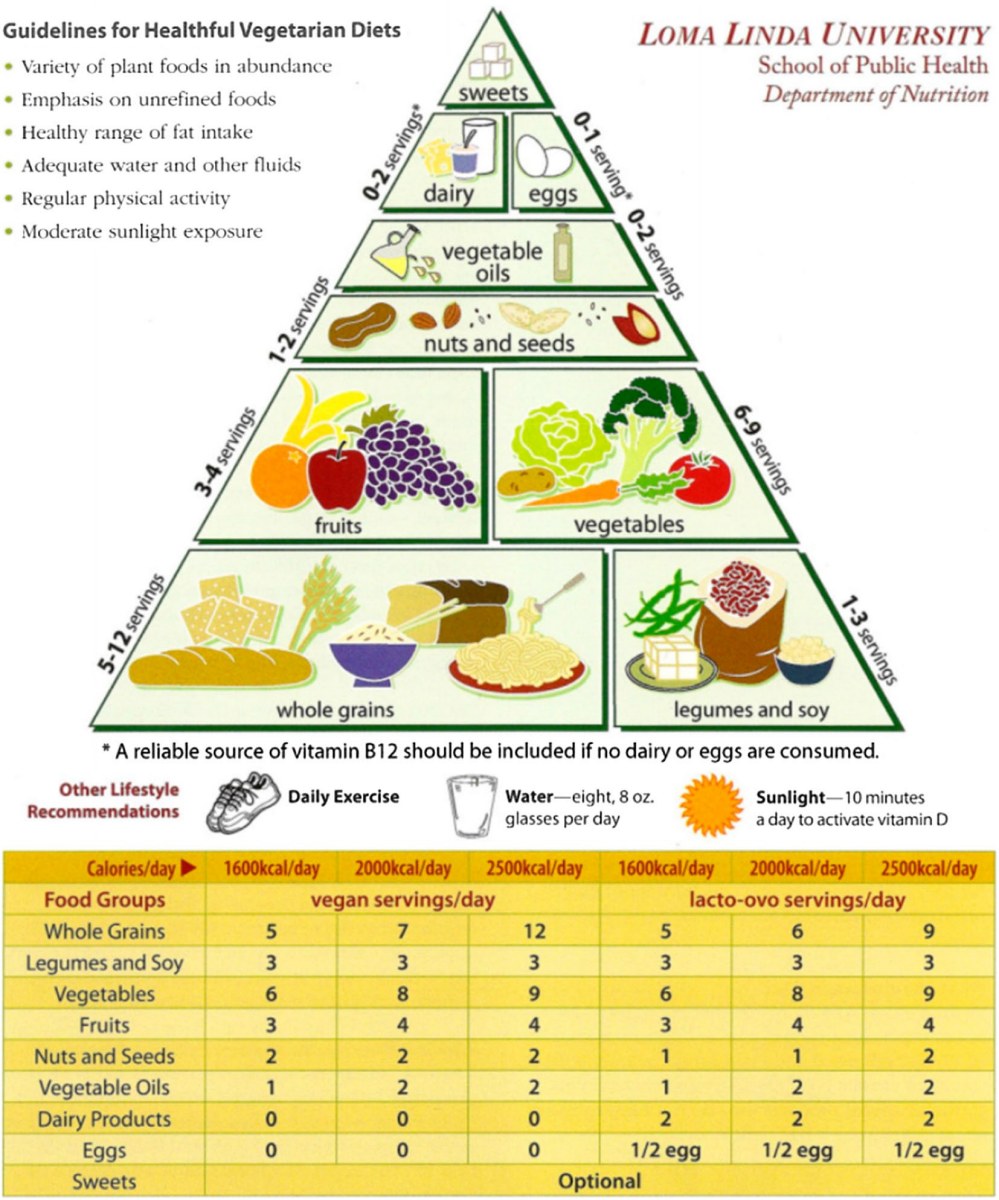
Vegetarian food guide pyramid guidelines for healthful vegetarian diets [21]. Note: We received the written permission from the authors for using the LLU Vegetarian Food Guide Pyramid in the present manuscript

Numerous prospective cohort studies and randomized clinical trials have shown the various health benefits of the vegetarian diet [21]. It is well known that a meat-free diet requires a well-balanced diet, including supplements or fortified products [22]. The Loma Linda University (LLU) Vegetarian Food Guide Pyramid [21] consists of both diet and lifestyle recommendations for a well-planned vegetarian diet (see Fig. 1).

Despite existing guidelines on a healthy vegetarian diet, the intake of a proper well-balanced and well-planed diet may prove difficult for some individuals following a meat-free diet. Research has suggested an association between vegetarianism and disordered eating behaviours (lifetime and current eating disorders) [3, 4]. Vegetarian diets may be used to legitimize food avoidance and avoidance of certain eating situations to facilitate ongoing restriction and disguise restrictive eating patterns employed to control weight [3]. The higher incidence of eating disorders relating to vegetarianism suggests that special diets (pescatarian, vegan, paleo, gluten-free and raw diet) may be connected to disordered eating behaviours and serve as socially acceptable means to mask disordered eating behaviours [4]. Research has shown that women following a vegetarian diet may be more likely to display disordered eating attitudes and behaviours than women following a meat diet [23] as well as men [24].

In addition, following specific diets or food rules, such as a vegetarian, vegan, fructarian (fruitarian) or crude diet (raw food diet), were found to be associated with orthorexic dietary patterns [2, 5–8]. A vegetarian or vegan diet might be a contributing factor for the onset of orthorexia nervosa. The permanent reduction of “allowed” foods might contribute to a diet that consists of very few foods considered comestible; consequently, individuals might restrict their diet from omnivore to vegetarian and finally to vegan [12].

**Fig. 2:**
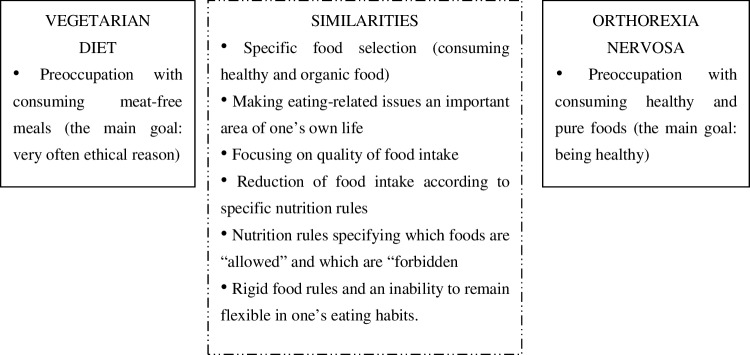
Similarities between vegetarian diet and orthorexia nervosa

Analysis of orthorexic eating behaviour reveals several overlapping characteristics with vegetarianism, veganism and dieting behaviour (see Fig. 2). It is worth pointing out that while there are several assumptions regarding the connection between a meat-free diet and orthorexic dietary patterns, there are no published data confirming those similarities [2].

Adherence to a vegetarian diet has been hypothesized to be a factor in the onset and maintenance of disordered eating behaviour; however, evidence to support this assumption has been largely inconsistent [25]. Some studies found that individuals following a vegetarian or vegan diet were more likely to have orthorexic eating behaviour than individuals on a mixed diet [4, 26, 27], while other results reported that those who followed a vegan diet presented a less pathologically strict health-oriented eating pattern than those following a meat diet [28], highlighting once again the gap in the literature on the relationship between a vegetarian diet and orthorexia nervosa. Although research on vegetarianism and veganism is becoming more prevalent, to date, only a few research studies have been conducted to explore this relationship. There has been only one study focusing on orthorexic and restrained eating behaviour in a sample of vegans and vegetarians [2]. The objective of the present study was to examine the orthorexic dietary patterns and eating behaviours among individuals with differential food preferences (vegetarian, vegan, and meat diet). In addition, we aimed to analyse the moderating role of ethical and health reasons for following a meat-free diet on the relationship between vegan versus vegetarian diet and eating behaviours and orthorexia nervosa. The assumption of the moderating role of reasons for following a meat-free diet was based on a recent research [2] showing that individuals who restrict their eating behaviour predominantly due to ethical reasons, display more orthorexic eating behaviour than individuals not limiting their food consumption. Moreover, in a sample of vegans, only health-related motives were associated with orthorexic eating behaviour, contrary to the ethical reasons. Those results indicate that motives and beliefs might moderate the effect of following meat-free diet on ON behaviours [2]. The current literature is lacking of research indicating predictors of ON in individuals following a meat-free diet. Therefore, this study aimed also to determine the predictors of cognitions, behaviours, and feelings related to orthorexia nervosa in individuals with differential food preferences.

On the basis of the literature [2], we put forward the following hypotheses:

- H1: Individuals following a vegetarian diet and/or a vegan diet present a higher level of orthorexic behaviours compared to the individuals following a meat diet.
- H2: Individuals following a vegan diet have a greater level of knowledge about healthy eating than those following a vegetarian diet. This effect is moderated by the reason for choosing a meat-free diet (ethical versus health).
- H3: Cognitive restraint is a predictor of a strict health-oriented eating pattern among individuals following a vegetarian and/or vegan diet.

## Materials and methods

### Participants following a vegetarian diet, a vegan diet, and control group

**Fig. 3:**
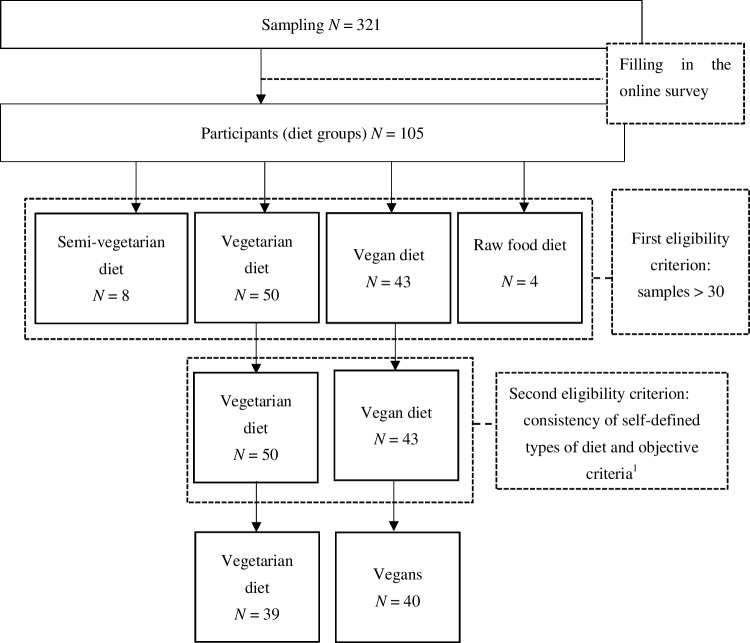
Eligibility criteria for the special diet sample research in the first study. Note: ^1^The reason participants were excluded due to “consistency of self-defined types of diet and objective criteria” was following: discrepancy between self-description of the diet and self-identification as one of the following: vegetarian or vegan (e.g., those who described themselves as vegetarians and declared to often eat fish were eliminated). The procedure was based on Barthels’ et al. [2] criteria: vegetarianism: exclusion of meat from the diet; veganism: exclusion of all animal products from the diet

The vegan and vegetarian sample was selected from 321 individuals following a meat-free diet who applied to participate in the study. Of this number, 105 individuals (32.71%) completed the online survey. This sample consisted of those following a semi-vegetarian diet (7.62%), a vegetarian diet (47.62%), a vegan diet (40.95%), and a raw food diet (3.81%). The eligibility criteria for the sample with a meat-free diet are presented in Fig. 3.

To divide each sample into subgroups, participants had to answer several questions regarding their eating behaviours (e.g., how often they eat red meat, poultry, fish and seafood, milk and dairy products, eggs, fruits, vegetables, grain products, and oil on a 5-point scale from ‘never’ to ‘every day’). Furthermore, participants were asked to identify themselves as one of the following: semi-vegetarian, vegetarian, vegan, fruitarian or raw food diet.

Referring to the recent research [2], which highlighted the important role of the reasons for beginning and preserving a vegetarian and vegan diet, we took into consideration these variables in our study. The sample was divided into two groups: ethical aspect (e.g., ethics, religion, value system, and environmental concerns) versus health causes (e.g., health and losing weight).

The control group consisted of 41 individuals following an omnivore diet (consumption of fruits, vegetables and animal products and meat).

### Procedure

Data of samples were collected via online survey. Participants were recruited via direct interpersonal contact (e.g., in vegetarian restaurants and vegetarian meetings), posters (about project), and vegetarian social networking sites. Participants from the control group were recruited using the same procedure in the places not linked to vegetarian/vegan lifestyle. All participants gave their permission to be part of the study, and they provided informed voluntary written consent prior to initiating the survey via an online consent form. Participants completed a series of measures (described below). They were informed that their participation was voluntary and anonymous. Furthermore, all participants had the right to refuse to participate without penalty if they wished (at any time and for any reason, they could refuse to answer a question or stop filling out the questionnaire and not send their data using the ‘send’ button). The touch pen (worth approximately €6.00) was compensation for participation in the research. No other compensation was offered. The study protocol was approved by the SWPS University of Social Sciences and Humanities Human Research Ethics Committee (no. WKEB45/03/2017). The research project was funded by the National Science Centre (NCN), Poland (Grant no. 2017/01/X/HS6/00007). The current study is part of a large project focusing on the assessment of rumination and eating behaviours in daily life among individuals with differential food preferences (following a meat-free diet).

### Measures

#### The Eating Habits Questionnaire (EHQ)

An challenge for research exploring the link between specific dietary habits and orthorexic behaviours is the valid evaluation of orthorexia nervosa. Bratman created the Orthorexia Self-Test, labelled by the author as “a ten-question quiz to determine if you have orthorexia” [12; p. 47]. The necessary psychometric properties, namely, reliability and validity, of this test were not evaluated. Moreover, neither the cut scores of a reference group was assessed [11]. It was designed as a screening instrument (as an informal measure), with items such as: ‘Do you spend more than 3 h a day thinking about healthy food?’ or ‘Does your diet socially isolate you?’. The Orthorexia Self-Test, however, has been the basis of the ORTO-15, ‘a questionnaire for the diagnosis of orthorexia nervosa’ [29, p. e28]. Nowadays, the ORTO-15 is probably the most widely used self-report measure of orthorexia nervosa. Although preliminary validation has shown that the ORTO-15 has good predictive validity [29], a low reliability has been ascribed, and the internal consistency of the ORTO-15 has been criticized [14]. There are many possible objections to the ORTO-15 test reliability [11, 26], e.g., high prevalence rates of ON among different research populations, lack of clearly articulated development of construct validity, lack of discussion about the creation of an item pool, lack of standardization methods, and lack the basic psychometric properties [11], suggesting caution in the usage of the ORTO-15 test to reliably measure the prevalence of ON. Dunn et al. [28] consider that the ORTO-15 likely cannot distinguish between healthy eating and pathologically healthful eating. In addition, according to Dunn and Bratman [10], the ORTO-15 is likely to measure healthy eating, but it is not feasible to more accurately and fully capture pathology. Therefore, taking into consideration all listed limitations, in the present study, we used the Eating Habits Questionnaire [13], a new research tool, developed independently of the ORTO-15, for the measurement of orthorexia nervosa.

The Eating Habits Questionnaire [13] assesses cognitions, behaviours, and feelings related to an extreme focus on healthy eating, which has been called orthorexia nervosa. This 21-item self-report inventory measures the following symptoms of orthorexia nervosa: (a) knowledge of healthy eating (5 items, e.g., ‘The way my food is prepared is important in my diet’; *α* = 0.90); (b) problems associated with healthy eating (12 items, e.g., ‘I have difficulty finding restaurants that serve the foods I eat’; *α* = 0.82); and (c) feeling positively about healthy eating (4 items, e.g., ‘I have made efforts to eat more healthily over time’; *α* = 0.86). In the present study, the EHQ was translated from English to Polish using a standard forward–backward translation procedure. The English version of the EHQ was first translated into Polish (by two translators who independently translated the same questionnaire) and then back-translated into English (by two independent native English speakers without reference to the English original). In the present study, the Cronbach’s *α* values of the three subscales were: 0.81 for knowledge of healthy eating, 0.82 for problems associated with healthy eating and 0.70 for feeling positively about healthy eating.

#### The Three-Factor Eating Questionnaire (TFEQ-R18)

The TFEQ-R18 measures eating behaviours [30]. It contains 18 items that constitute 3 domains: cognitive restraint (6 items, e.g., ‘I consciously hold back at meals in order not to weight gain’), uncontrolled eating (9 items, e.g., ‘Sometimes when I start eating, I just can’t seem to stop’), and emotional eating (3 items, e.g., ‘When I feel blue, I often overeat’). In the present study, we used the Polish version of the questionnaire [31], which has demonstrated satisfactory levels of internal reliability (*α* = 0.78 for cognitive restraint, *α* = 0.84 for uncontrolled eating and *α* = 0.86 for emotional eating). In the present study, the Cronbach’s *α* values of the three subscales were: 0.77 for cognitive restraint, 0.86 for uncontrolled eating and 0.88 for emotional eating.

### Data analysis

The Statistical Package for Social Sciences (version 22.0) was used for variance, moderating and regression analysis. One-way ANOVA with factor group for independent samples (vegetarian diet versus vegan diet versus control group) was taken, and post hoc tests with Bonferroni correction were used. The PROCESS macro [32] with bootstrap *N* = 10,000 was used to analyse the moderating effects. Subsequently, a multiple linear regression was used for analysis of the predictors in samples with a meat-free diet (vegetarians and vegans groups).

## Results

### Participant characteristics

Detailed characteristics of participants are presented in Table 2.

**Table 2:** Sample characteristics

	Vegetarian diet *N* = 39	Vegan diet *N* = 40	Omnivore diet *N* = 41
*M* (SD)			
Age	26.54 (8.07)	29.72 (10.75)	30.27 (10.04)
Body mass index (kg/m^2^)	21.78 (2.45)	21.72 (4.09)	23.11 (7.01)
Duration of diet (in months)	76.20 (105.15)	45.95 (66.17)	n/a
*N* (%)			
Body mass index			
Underweight	2 (5.13)	6 (15.00)	4 (9.76)
Normal body			
Weight	35 (89.74)	27 (67.50)	26 (63.41)
Overweight	2 (5.13)	7 (17.50)	6 (14.63)
Obesity	0 (0)	0 (0)	5 (12.20)
Weight loss methods			
Yes			
Diet	3 (7.69)	3 (7.50)	4 (9.76)
Physical activity	9 (23.08)	6 (15.00)	10 (24.39)
Laxatives	0 (0)	0 (0)	0 (0)
Vomit	0 (0)	0 (0)	0 (0)
Starvation diet	0 (0)	0 (0)	0 (0)
No	27 (69.23)	31 (0)	27 (65.85)
Daily weighing			
Yes	0 (0)	1 (2.50)	4 (9.76)
No	39 (100)	39 (97.50)	37 (90.24)

There were no differences between the vegan and vegetarian sample and the control group (following an omnivore diet) in terms of gender [*F*(2,117) = 2.33, *p* > 0.05, *η*^2^ = 0.038], age [*F*(2,117) = 1.71, *p* > 0.05, *η*^2^ = 0.028] and body mass index [*F*(2,117) = 1.03, *p* > 0.05, *η*^2^ = 0.017].

### Variance analysis: orthorexia nervosa and eating behaviours

**Fig. 4:**
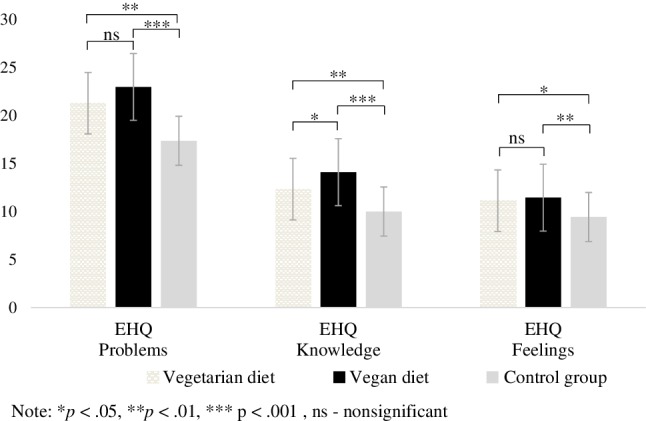
Means and standard error of the symptoms of orthorexia nervosa as measured by the Eating Habit Questionnaire (EHQ)

The results of the one-way ANOVA with group (vegan, vegetarian, and control) as an independent variable and EHQ dimensions as the outcome indicates that there is a significant group difference in orthorexia nervosa, especially in the particular dimensions linked to healthy eating, *F*(2,117) = 11.59, *p* < 0.001, *η*^2^ = 0.165; knowledge of healthy eating, *F*(2,117) = 19.35, *p* < 0.001, *η*^2^ = 0.249 and feeling positively about healthy eating, *F*(2,117) = 6.42, *p* < 0.01, *η*^2^ = 0.099 (see Fig. 4).

**Fig. 5:**
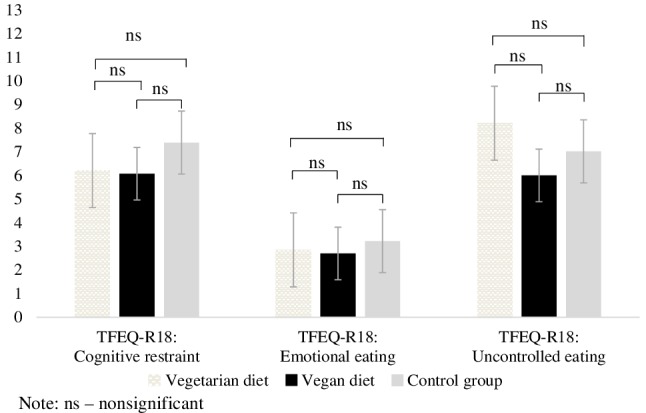
Means and standard error of the eating behaviours as measured by the Three-Factor Eating Questionnaire-R18 (TFEQ-R18)

There were no significant differences between the groups in the dimensions of the TFEQ-18, namely: cognitive restrain, *F*(2,117) = 1.60, *p* > 0.05, *η*^2^ = 0.027; emotional eating, *F*(2,117) = 0.350, *p* > 0.05, *η*^2^ = 0.006 and uncontrolled eating, *F*(2,117) = 1.58, *p* > 0.05, *η*^2^ = 0.026 (see Fig. 5).

### Moderating role of the reason for following a specific diet

**Fig. 6:**
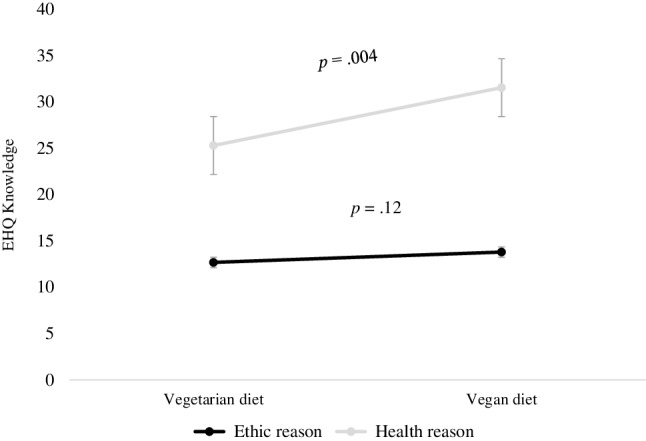
Moderation effect of reasons for following specific diet

To explore the moderating role of the reason for following a vegetarian diet as suggested by Barthels et al. [2], we performed a series of moderation models using Process plug-in software for SPSS [32]. The lower level confidence interval and the upper-level confidence interval for unconditional effects are presented in square brackets. This method of moderation analysis was chosen because of the unequal distribution of the participants in the variable reason for following a meat-free diet (health versus ethics) and, consequently, a lack of satisfying assumptions for performing the analysis of variance. The results suggest a significant moderation model with the type of diet (vegetarian versus vegan) as a predictor, reasons for following this diet as a moderator and EHQ knowledge of healthy eating as an outcome variable (*R* = 0.39; *F*(3,71) = 4.19; *p* < 0.008; MSE = 7.98). The conditional effect of diet type on EHQ knowledge of healthy eating was significant for participants following the diet for health-related reasons (*B* = 2.56; [0.84, 4.28]; *p* = 0.004); this effect was not significant for participants following a meat-free diet for ethical reasons (*B* = 0.56; [− 0.15, 1.27]; *p* = 0.12). The moderation effect is presented in Fig. 6. The conditional process models with other dimensions of the EHQ or the TFEQ-18 as outcomes were not significant.

### Regression analysis: orthorexia nervosa and eating behaviours among individuals following a meat-free diet

The predictor analysis of an extreme focus on healthy eating indicates significant models for all symptoms of orthorexia nervosa (entered into the model separately): problems associated with healthy eating, *F*(6,72) = 4.12, *p* < 0.001, knowledge of healthy eating, *F*(6,72) = 3.29, *p* < 0.01 and feeling positively about healthy eating, *F*(6,72) = 5.22, *p* < 0.001 (see Table 2). A multiple linear regression was used to analyse the predictors. The dichotomous variable (vegan versus vegetarian diet) was subjected prior to analysis (Table 3).

**Table 3:** Prediction models of orthorexia nervosa in individuals following a meat-free diet

Variable	*β*	*R*^2^-change
Model 1: Problems associated with healthy eating		
Cognitive restraint	0.411***	0.193
Emotional eating	− 0.062	
Uncontrolled eating	− 0.065	
Type of diet^a^	0.166	
Duration of diet	− 0.092	
Body mass index	− 0.222*	
Model 2: Knowledge of healthy eating		
Cognitive restraint	0.331**	0.150
Emotional eating	− 0.099	
Uncontrolled eating	− 0.050	
Type of diet^a^	0.292**	
Duration of diet	0.087	
Body mass index	− 0.167	
Model 3: Feeling positively about healthy eating		
Cognitive restraint	0.420***	0.245
Emotional eating	− 0.101	
Uncontrolled eating	0.161	
Type of diet^a^	0.055	
Duration of diet	− 0.233*	
Body mass index	− 0.165	

*β* the standardized beta

*p < 0.05, **p < 0.01, ***p < 0.001

^a^Vegetarian and vegan diet

## Discussion

The first objective of the present study was to assess the orthorexic dietary patterns and eating behaviours among individuals with differential food preferences. Our results suggested that individuals who followed a special diet (vegetarian and vegan diet) reported more orthorexic behaviours (knowledge of healthy eating, problems associated with healthy eating and feeling positively about healthy eating) than those who followed no special diet; thus, Hypothesis 1 was confirmed. An extreme focus on healthy eating is associated with special eating behavioural features: vegetarian and vegan diet. Our results are compatible with the findings of Barnett et al. [4]. The recent study has also shown that a meat-free diet, lower educational attainment and higher depressive symptoms were associated with a higher rate of orthorexic behaviour [33]. Vegetarians and vegans exhibit higher ON tendencies than individuals on a mixed diet, which may indicate that individuals with ON tendencies are more likely to be on a vegetarian or vegan diet [26]. The authors argue that being on a vegetarian diet requires a fair degree of self-discipline, planning, and cognitive processing related to eating behaviour. In the literature, we can also find results indicating no significant difference in an extreme focus on healthy eating (also in attitudes to eating and obsessive symptoms) between the individuals following a vegetarian or vegan diet and individuals consuming meat [34]. Thus, Çiçekoğlu and Tunçay [34] state that veganism and/or vegetarianism is not associated with an obsession with healthy eating.

Our study showed that individuals who followed a vegetarian or vegan diet did not differ in problems associated with healthy eating and feeling positively about healthy eating. Thus, there is no difference between these groups in turning down social events that involve eating unhealthy food; following a diet with many rules; being distracted by thoughts of eating healthily; eating only what their diet allows; considering their healthy eating as a source of stress in their relationship; considering their diet affects the type of employment they would take; having difficulty finding restaurants that serve the foods they eat; having few foods that are healthy for them to eat; going out less, since they began eating healthily and spending more than 3 h a day thinking about healthy food and following a health-food diet rigidly (items associated with problems with healthy eating). In addition, the study did not show significant between-group differences in terms of making efforts to eat more healthily over time, feeling in control when they eat healthily, feeling a sense of satisfaction in eating the way they do and feeling great when they eat healthily (items associated with feeling positively about healthy eating). This would suggest that individuals following a vegetarian and vegan diet presented similar problems associated with healthy eating and similar patterns of feeling positively about healthy eating. It may also suggest that both groups have an interest in (or they are preoccupied with) healthy eating comparing to individuals following a meat diet. Our findings suggest that following a special diet could prompt more focus on the quality of foods consumed both in individuals following a vegetarian diet and individuals following a vegan diet.

The conclusions of our paper are in line with the results of the latest research [2], which have shown that vegetarians and vegans do not differ in orthorexic eating behaviour, but both groups presented higher level of orthorexic eating behaviour than individuals with rare and frequent meat consumption. Moreover, individuals showing restrained eating behaviour mainly because of ethical reasons or with the aim to lose weight, present more orthorexic eating behaviour than those who do not limit their food intake. The authors [2] argue that a vegan diet does not directly result in a disordered eating behaviour, nevertheless, the prevalence of ON in the vegan (7.9%) and vegetarian groups (3.8%) are higher than in the individuals consuming meat (3.6% of participants with rare meat consumption and 0% of participants with frequent meat consumption). This could point to the fact that vegetarian or vegan diet could increase the risk of ON [2]. Unpublished research [in 2] has shown that in vegans, ON is solely related to health-related motives, whereas ethical reasons are not, indicating that underlying motives and beliefs might moderate this effect. Other studies [35] have shown that individuals who follow a vegetarian diet (ashtanga practitioners) present more pathological symptoms of strict health-oriented eating patterns (the prevalence rate for orthorexia in this group was 43%), and they might push their attention to it to potentially orthorexic limits.

Our results also demonstrated that individuals who focus on excluding all animal products (meat, seafood, poultry, eggs and dairy) from their daily diet showed a higher level of knowledge of healthy eating than those who followed a vegetarian diet (Hypothesis 2 was confirmed) and those who followed an omnivore diet (Hypothesis 1 was confirmed). They are more informed than other individuals with differential food preferences about healthy eating, and the way their food is prepared is more important in their diet than in those following a vegetarian or omnivore diet. In their opinion, their eating habits are superior, and their diet is better than other individuals’ diets. They are also convinced that they prepare food in the most healthy way. A vegan diet might become a guise for disordered eating, including for orthorexia nervosa, and might provide an excuse for following food rules that result in the removal of whole food groups [36].

In the present study, we also aimed to analyse the impact of ethical and health reasons for following a meat-free diet on the relationship between vegan versus vegetarian diets and eating behaviours. Participants maintaining a meat-free diet for health reasons had more risk on the knowledge subscale of EHQ, but this effect was significant only for vegans (for vegetarians, there were no difference between ethical and health reasons) (Hypothesis 2 was confirmed). Therefore, vegans were a group more likely to develop cognitive orthorexic eating behaviours (knowledge subscale) if they were on a meat-free diet for health reasons. So, ethical causes might be a protective factor in the development of orthorexic eating behaviours as a cognitive aspect in this group.

The third objective of the present study was to identify the predictors of cognitions, behaviours and feelings related to orthorexia nervosa among individuals with differential food preferences. Our research showed that cognitive restraint was a predictor of orthorexia nervosa among a sample following a meat-free diet (vegetarian and vegan diet) (Hypothesis 3 was confirmed). Focusing on the control of food intake might start out as a claim for healthy eating and advance into increasingly restrictive dietary rules [36]. The consequences of cognitive restraint (stable disposition to limit and control food intake) could be following: dysregulation of internal perceptions of hunger and satiety (which are essential for homeostatic regulation), emotional dysregulation, low self-esteem and low body satisfaction [37]. Martins et al. [38] report that vegetarianism might be used as a mask for dieting behaviour. The recent study has shown that individuals with a special diet self-reported significantly more current and past eating disorders compared to those following no special diet [4].

It is worth pointing out that our study also showed that besides cognitive restraint, (a) lower (reduced) body mass index determines problems associated with healthy eating, (b) following a vegan diet is a predictor of knowledge of healthy eating, especially among those who follow a vegan diet for health reasons and (c) shorter duration of following a vegetarian and vegan diet predicts feeling positively about healthy eating among individuals following a meat-free diet (vegetarian and vegan diet). Therefore, there are reasons to suspect that cognitions connected with an extreme focus on healthy eating could be related to control weight or weight loss, which was previously suggested in other studies [2, 3, 11]. Focusing on diet based on the complete exclusion of all animal-based products results in adopting strategies to substitute animal protein-dense foods with plant protein-dense foods and plant-based food products (e.g., textured soy products, almond, rice milk, uncooked cereals, seeds) along with an increase in the consumption of meat substitute foods [39]. The fact that vegans’ daily food intake is very selective could explain their advanced knowledge of healthy eating. In case of feelings linked to orthorexia nervosa, our findings could suggest that early identification of following a vegetarian and vegan diet may be an important factor in preventing orthorexic behaviours. It could also indicate that restrictive eating might be a regulator of emotional state—especially in situations associated with high levels of anxiety and guilt after eating high-caloric foods.

It is worth paying attention to other studies which indicate behavioural and cognitive features associated with ON (e.g., weigh lost, less pathological body image discomfort) [10, 15] and its relationship with eating disorders (e.g., anorexia nervosa, AFRID) [10, 40–42] as well as with other psychopathological dimensions (e.g., obsessive–compulsive disorder, obsessive–compulsive personality disorder) [14, 40]. Study [18] on the brain–behaviour relationship has shown that ON was independently related to executive function deficit (cognitive rigidity, emotional control, self-monitoring and working memory) which AN and OCD profiles already overlap.

Several limitations in the present study should be acknowledged. First, our sample size did not include a large number of individuals who followed a special diet, which may have reduced our ability to find significant differences among special diet groups. Second, our sample cannot be considered representative for all individuals following a vegetarian and vegan diet; it would be desirable to replicate and extend the present findings in the future. Third, we evaluated a posteriori the reason for following a meat-free diet, and the number of individuals choosing ethical versus health reasons was not equivalent. Fourth, we did not assess emotional distress related to food choices. Emotional distress might have a significant impact on ON, as disordered eating behaviours might be considered as emotional regulation strategy [43]. Consequently, this variable should be taken into account as potential moderator of the link between specific dietary habits and ON in the further research. However, it is important to underline that linking participants distress to ON behaviour would require assessing those variables with ambulatory assessment (contrary to for example the reason for following the diet that changes over time less dynamically that participants’ distress) and the present research by exploring the link between type of diet, ON behaviour and potential mediators (like reasons for following the diet) provides the basis for further development of this kind of ambulatory research. Forthcoming research should include also larger sample sizes and simple random sampling across the general population (among individuals indicating an omnivore diet). Besides, the current results are based on self-reporting that could be subject to potential bias. Moreover, the current study was a cross-sectional one and could not assess the causality of relationships. Future studies should determine causal relationships between measured variables and potential mediators in ambulatory settings.

Despite the aforementioned limitations, our results could contribute to identify potential risk factors for strict health-oriented eating patterns and to gain a better insight into orthorexia nervosa. We suppose that following diet and lifestyle recommendations for a well-balanced vegetarian diet (see Fig. 1) could be helpful for individuals with orthorexic eating behaviours to better plan both the quality and quantity of their meals.

Further research is needed to investigate whether vegetarianism and/or veganism serve as risk factors for developing orthorexic eating behaviours. It is also worth examining vegetarianism and orthorexic eating behaviour longitudinally to better understand how orthorexia nervosa symptoms and vegetarianism may propel each other over time.

Expanding the knowledge about ON will contribute to both public health and clinical research. In public health research it might help in developing prevention programs addressing orthorexic eating behaviour. In clinical research, it contributes to assess a much needed therapeutic program for the treatment of ON. The research providing a general knowledge on ON and dietary behaviours enables also to determinate more precisely crucial variables to measures or manipulate in the further studies.
